# Esophageal tuberculosis as an unusual cause of dysphagia: a case report

**DOI:** 10.11604/pamj.2022.41.225.29790

**Published:** 2022-03-18

**Authors:** Moufida Mahmoudi, Samir Bradai, Amal Khsiba, Asma Ben Mohamed, Jawhar Bradai, Mouna Medhioub, Lamine Hamzaoui, Mohamed Mousadek Azouz

**Affiliations:** 1 Gastroenterology Department, Mohamed Tahar Maamouri Hospital, Nabeul, Tunisia,; 2 Radiology Department, Mohamed Tahar Maamouri Hospital, Nabeul, Tunisia

**Keywords:** Dysphagia, tuberculosis, esophageal localization, case report

## Abstract

Esophageal tuberculosis is a rare cause of infectious esophagitis, even in countries with endemic tuberculosis. This impairment is often secondary. We report a case of secondary esophageal tuberculosis in an immunocompetent patient, clinically revealed by dysphagia. Esophagogastroduodenoscopy showed a large ulcer in the middle third of the esophagus with a fistula opening in the center of the ulcer. Histopathological examination of multiple esophageal tissue biopsies revealed epithelioid cell granulomas without caseous necrosis. We completed with Computed Tomography (CT) scan of the chest which revealed a fistula of the middle third of the esophagus, multiple mediastinal necrotic adenopathies and diffuse pulmonary micronodules suggesting miliary tuberculosis. Sputum examination for acid-fast-bacilli was positive. Anti-tuberculosis treatment resulted in a good response with complete remission. It is therefore important to recognize and include this entity in the differential diagnosis of patients with dysphagia particularly in countries with a high incidence of tuberculosis.

## Introduction

Extra pulmonary tuberculosis represents 15% of all tuberculosis (TB) [1]. The involvement of the digestive tract is dominated by peritoneum and ileocecal location [2]. Esophageal TB is a rare condition and it constitutes 0.3-2,8% of all gastrointestinal TB cases [3,4]. It usually occurs as a result of direct extension from mediastinal nodes (rarely from the lungs or bloodstream) or more rarely due to primary involvement of the esophagus [5]. Dysphagia as a presenting manifestation of TB is even more uncommon. Delay in diagnosis leads to the occurrence of several complications. We report a case of secondary esophageal TB revealed by dysphagia and we performed a literature review regarding this rare condition.

## Patient and observation

**Patient information:** a 55-year-old woman without medical history presented to the hospital with progressive dysphagia, primarily for solid foods then for both liquids and solids. The patient reported 10 kg weight loss, loss of appetite and intermittent fever. She did not report chest pain, dyspnea, coughing episodes, nausea, vomiting or hematemesis.

**Clinical findings:** the clinical examination found a temperature of 37.8°C, blood pressure at 130/80 mm Hg, a heart rate of 80 beats per minute. The abdomen was soft without any palpable masses or hepatosplenomegaly. The rest of the examination was normal.

**Timeline of the current episode:** the symptomatology began 3 months before her admission.

**Figure 1:**
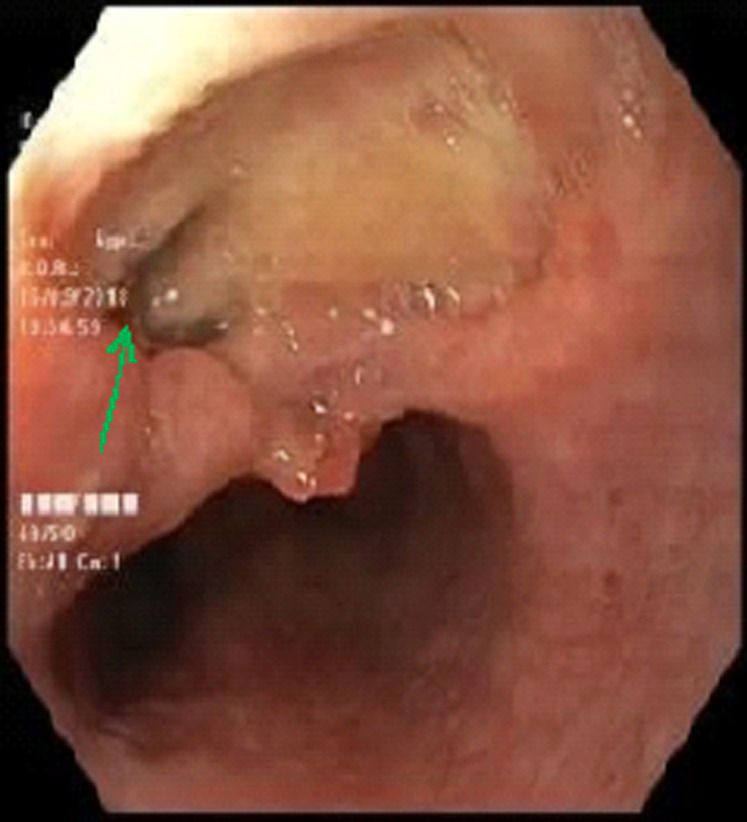
esophageal ulcer with a fistula opening (green arrow)

**Figure 2:**
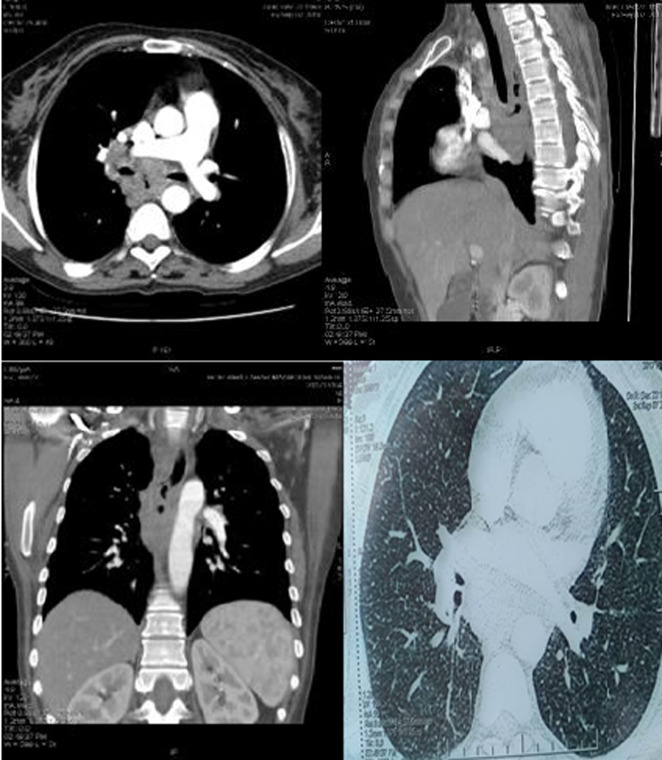
CT-scan findings: esophago-mediastinal fistula, mediastinal adenopathies and military tuberculosis

**Diagnostic assessment:** blood investigations revealed lymphocytosis with an elevated erythrocyte sedimentation rate. Esophagogastroduodenoscopy (EGD) showed a large ulcer measuring 3 cm in diameter in the middle third of the esophagus with a fistula opening in the center of the ulcer (Figure 1). Multiple biopsies were taken from the lesion. Histopathological examination revealed epithelioid cell granulomas without caseous necrosis. No evidence of malignancy and no acid-fast bacilli have been seen. We completed with computed tomography (CT) scan of the chest which revealed a fistula of the middle third of the esophagus without signs of mediastinitis, multiple mediastinal necrotic adenopathies and diffuse pulmonary micronodules suggesting miliary tuberculosis (Figure 2). Sputum examination for acid-fast-bacilli was positive. The patient tested negative for Human immunodeficiency virus (HIV).

**Diagnosis:** the diagnosis of miliary tuberculosis with secondary esophageal involvement in an immunocompetent patient was retained.

**Therapeutic interventions:** we started the treatment combining isoniazid, rifampicin, pyrazinamide, and ethambutol for 2 months initially and followed by a period of six months with two drugs (isoniazid and rifampicin).

**Follow-up and outcome of interventions:** the clinical course was favorable with weight gain and resolution of the dysphagia after three months from the start of treatment. The EGD was carried out after stopping treatment. It revealed the cicatrization of the esophageal ulcer. Computed tomography scan showed the regression of the mediastinal and pulmonary involvement. The patient has been followed up regularly and she is currently doing well, two years after the diagnosis.

**Patient perspective:** she was satisfied with the diagnostic and the proposed care.

**Patient consent:** she has given her consent for her images and other clinical informations to be reported in the journal. The patient understands that her name and initials will not be published.

## Discussion

Esophageal TB is a rare condition and accounts for 0.3-2.8% of all cases of gastrointestinal tuberculosis [3,4]. Tuberculosis can involve the esophagus, either as a primary infection or as a secondary manifestation of reactivated disease in most cases [6,7]. The esophageal involvement is usually secondary to contiguous extension from adjacent structures, such as mediastinal lymph nodes [6,7]. Other mechanisms may explain the extension of the infection to the esophagus through ingestion of infected sputum, hematogenous spreading in the case of disseminated miliary TB and retrograde extension from lymphatic drainage [2].

Dysphagia is the most common presenting symptom which occurs in about 90% of the cases [8]. The occurrence of symptoms such as odynophagia, retrosternal pain, fever, weight loss and anorexia have also been reported [2]. Given the rarity of the location, few cases have been reported where dysphagia revealed tuberculosis [5]. Complications of the esophageal involvement include bleeding, perforation, aspiration pneumonia, hematemesis, traction diverticula, esophageal strictures and fistula formation [9,10].

Esophagogastroduodenoscopy is the first examination performed in case of dysphagia and it allows doing biopsies. The endoscopic findings can range from esophageal ulcer or many nodules to a hypertrophic growth as an esophageal polyp or tumor like lesions. Esophagogastroduodenoscopy can find also a fistula opening, stricture or an aspect of extrinsic compression [2,9]. It can involve any segment of the esophagus, but most often involves the middle third at the level of the carina [5].

Histopathology and TB- polymerase chain reaction (PCR) remain the investigations of choice for confirming the diagnosis of esophageal TB [2]. Histology shows epithelioid granuloma with Langhans cells and central caseous necrosis [2]. But, the sensitivity of identifying typical caseating granulomas or Acid-fast bacillus staining on histopathology of endoscopy samples is low [2,6]. In fact, caseating granulomas are located deep in the submucosal layer of esophagus, multiple and deep esophageal endoscopic biopsies should be performed [6,7]. Tuberculosis-polymerase chain reaction (TB-PCR) has an important diagnostic interest when the biopsies are non-conclusive [11]. CT scan with contrast is also a useful tool, and particularly helps to search characteristic tuberculous lymphadenitis and also allows the search for other locations of TB [9,12]

Given its rarity and non-specific symptoms, clinician must be aware of this diagnosis to avoid misdiagnosis and treatment delay. The differential diagnosis of esophageal TB includes esophageal carcinoma, Crohn´s disease, moniliasis, actinomycosis, syphilis, and esophageal injury secondary to the ingestion of caustic material [2]. Esophageal TB, even complicated form with esophago-tracheal and esophago- mediastinal fistulas is primarily managed medically with antituberculosis therapy, as in our case [2,12]. The treatment combining isoniazid, rifampicin, pyrazinamide, and ethambutol is indicated for 2 months initially and then followed by a period of four to six months with two drugs (isoniazid and rifampicin) [5]. Endoscopic and surgical treatments are reserved to cases of complications, such as bleeding aorto-esophageal fistula, stricture or tracheo-esophageal fistula [5,12]. Complications are very rare and can be effectively avoided with a complete course of antituberculosis therapy on time and the earliest possible [6].

## Conclusion

The involvement of the esophagus in TB is uncommon. This location may reveal disseminated tuberculosis. Dysphagia is the most common symptom of esophageal TB. Given its rarity and resemblance with other esophageal pathologies; the clinical, radiological and endoscopic features of esophageal TB are not well defined. It is therefore important to recognize and include this entity in the differential diagnosis of patients with dysphagia particularly in countries with a high incidence of TB.
